# Prolonged Cannabidiol Treatment Effects on Hippocampal Subfield Volumes in Current Cannabis Users

**DOI:** 10.1089/can.2017.0047

**Published:** 2018-04-01

**Authors:** Camilla Beale, Samantha J. Broyd, Yann Chye, Chao Suo, Mark Schira, Peter Galettis, Jennifer H. Martin, Murat Yücel, Nadia Solowij

**Affiliations:** ^1^ School of Psychology, Illawarra Health and Medical Research Institute, University of Wollongong, Wollongong, Australia.; ^2^ Brain and Mental Health Laboratory, Monash Institute of Cognitive and Clinical Neurosciences, School of Psychological Sciences, Monash University, Clayton, Australia.; ^3^ Discipline of Clinical Pharmacology, School of Medicine and Public Health, University of Newcastle, Callaghan, Australia.; ^4^ The Australian Centre for Cannabinoid Clinical and Research Excellence (ACRE), New Lambton Heights, Australia.

**Keywords:** CA1, cannabidiol, cannabis, hippocampal subfields, hippocampus, subiculum

## Abstract

**Introduction:** Chronic cannabis use is associated with neuroanatomical alterations in the hippocampus. While adverse impacts of cannabis use are generally attributed to Δ^9^-tetrahydrocannabinol, emerging naturalistic evidence suggests cannabidiol (CBD) is neuroprotective and may ameliorate brain harms associated with cannabis use, including protection from hippocampal volume loss. This study examined whether prolonged administration of CBD to regular cannabis users within the community could reverse or reduce the characteristic hippocampal harms associated with chronic cannabis use.

**Materials and Methods:** Eighteen regular cannabis users participated in an ∼10-week open-label pragmatic trial involving daily oral administration of 200 mg CBD, with no change to their ongoing cannabis use requested. Participants were assessed at baseline and post-CBD treatment using structural magnetic resonance imaging. Automated longitudinal hippocampal segmentation was performed to assess volumetric change over the whole hippocampus and within 12 subfields.

**Results:** No change was observed in left or right hippocampus as a whole. However, left subicular complex (parasubiculum, presubiculum, and subiculum) volume significantly increased from baseline to post-treatment (*p*=0.017 uncorrected) by 1.58% (Cohen's *d*=0.63; 2.83% in parasubiculum). Heavy cannabis users demonstrated marked growth in the left subicular complex, predominantly within the presubiculum, and right cornu ammonis (CA)1 compared to lighter users. Associations between greater right subicular complex and total hippocampal volume and higher plasma CBD concentration were evident, particularly in heavy users.

**Conclusions:** Our findings suggest a restorative effect of CBD on the subicular and CA1 subfields in current cannabis users, especially those with greater lifetime exposure to cannabis. While replication is required in a larger, placebo-controlled trial, these findings support a protective role of CBD against brain structural harms conferred by chronic cannabis use. Furthermore, these outcomes suggest that CBD may be a useful adjunct in treatments for cannabis dependence and may be therapeutic for a range of clinical disorders characterized by hippocampal pathology (e.g., schizophrenia, Alzheimer's disease, and major depressive disorder).

## Introduction

Regular and prolonged cannabis use has been associated with morphological^1,2^ and functional brain changes,^2–4^ cognitive impairment,^5^ and increased risk of adverse mental health outcomes, including the precipitation of psychotic symptoms and disorders.^6,7^ Neuroimaging evidence has consistently identified the hippocampus, a subcortical brain region critically involved in learning and memory and implicated in psychopathology, as a particular locus of compromise.^1,2^ Long-term heavy cannabis users have demonstrated reduced hippocampal volume^8,9^ and gray matter density^10^ compared to nonuser controls, with some evidence for greater volume loss resulting from greater exposure to cannabis.^1^ Alterations to hippocampal shape,^11^ neurochemistry,^8^ and structural^12^ and functional connectivity^13^ in chronic cannabis users have also been reported. Recently, we showed that volumetric reduction of specific hippocampal subfields (cornu ammonis [CA]1–4 and dentate gyrus [DG]) was sensitive to cannabis dependence, with an inverse correlation between greater lifetime cannabis exposure and subfield volumes in dependent, but not nondependent users.^14^

Cannabinoid type 1 receptors (CB1Rs) are abundant throughout the brain, but occur in high density in specific regions, including the hippocampus.^15^ Cannabis-related neurobiological, cognitive, and psychological harms are generally ascribed to Δ^9^-tetrahydrocannabinol (THC), the primary psychoactive constituent of cannabis, which is a partial agonist at CB1Rs.^16,17^ Acute administration of THC to humans dose-dependently transiently increases anxiety, impairs cognition, and induces a range of positive and negative psychotic-like symptoms, including paranoia, delusions, and conceptual disorganization.^18–20^ While the precise mechanisms underlying these effects are not well understood, animal studies have shown that THC accumulates in neurons,^21^ with long-term exposure to THC resulting in neurotoxic changes in hippocampal microstructure.^22^ Conversely, cannabidiol (CBD) is the second most abundant cannabinoid in cannabis and has purported neuroprotective,^23^ anxiolytic,^24^ and antipsychotic^25^ properties. Acutely administered, CBD exerts opposing effects to THC upon activation of specific brain regions, including the hippocampus,^26^ and ameliorates the induction of hippocampal-dependent cognitive impairment and psychotic-like symptoms by THC in healthy volunteers.^27^ CBD has a low affinity for CB1Rs, yet demonstrates the capacity to antagonize CB1R agonists, which may underlie its functional antagonism of THC.^28^ Preclinical studies have shown CBD to induce synaptic plasticity and facilitate hippocampal neurogenesis,^29,30^ with some evidence suggesting that the proneurogenic action of CBD via the hippocampus may underlie its anxiolytic effects.^30^ Although precise neurobiological mechanisms by which CBD may promote neurogenesis remain unclear, modulation of endocannabinoids such as anandamide through CB1Rs has been implicated.^29,31^

In recent years, increasingly potent strains of cannabis containing high levels of THC and decreasing levels of CBD have dominated the market, raising concerns for greater THC-related harms in the community.^7,32^ Naturalistic studies examining proportional exposure to THC and CBD by hair analysis of chronic cannabis users found that the presence of CBD in cannabis was associated with fewer psychotic-like symptoms,^33,34^ improved recognition memory,^34^ and increased hippocampal gray matter concentration.^10^ We have recently shown apparent normalization of hippocampal volume and n-acetylaspartate (NAA; a marker of hippocampal neuronal integrity) levels in cannabis users regularly smoking cannabis containing CBD, such that they were indistinguishable from controls, while those exposed to THC but not CBD, showed 11% smaller hippocampal volumes and 15% lower NAA concentrations than controls.^8^ These findings indicate CBD may protect against neurobiological and psychological harms of regular cannabis use; however, the cross-sectional nature of these studies precludes any inferences about directionality.

The possibility that prolonged administration of CBD to cannabis users may protect against or reduce THC-induced harms is intriguing. The hippocampus is highly neuroplastic and volume growth has been demonstrated following relatively brief interventions (e.g., increase in hippocampal volume by 12% in patients with schizophrenia and 16% in healthy controls following 12 weeks of aerobic exercise,^35^ indicating this is an adequate timeframe to observe discernible treatment effects in the hippocampus). Hence, this study aimed to investigate whether prolonged administration of CBD may reverse hippocampal volumetric reduction typically observed in long-term, heavy cannabis users, but recruitment was broadened to enable examination of effects of CBD treatment in a sample with a range of cannabis experience. An ∼10-week pragmatic, open-label trial was undertaken, in which cannabis users within the community consumed 200 mg CBD in capsule form daily, in the context of their ongoing cannabis use. Structural magnetic resonance imaging (MRI) scans were completed at baseline and post-CBD treatment. It was hypothesized that prolonged CBD administration would result in increased hippocampal volume at post-treatment, as subserved by growth in specific subfields such as CA1, subiculum, and DG, due to the high concentration of CB1Rs in these subregions^15^ and their involvement in neurogenesis.^36^

## Materials and Methods

### Participants

Twenty regular cannabis users (at least once per month for a minimum 6 months) aged 18–55 years were recruited from the community through newspaper and online advertisements. Exclusion criteria were current or past regular (greater than once per month for >6 months in the past 3 years) other illicit drug use or dependence on or treatment-seeking for any substance other than cannabis; history of synthetic cannabinoid use; any neurological disorder or serious head injury; psychiatric history (assessed by the Mini-International Neuropsychiatric Interview Plus^37^) or medication use; pregnancy or lack of contraception for female cannabis users; and contraindications for MRI. Eighteen participants completed both baseline and post-treatment MRI scans and are reported in this study. Participants provided informed written consent at baseline, post-treatment, and each weekly session (see Procedure, CBD administration, and dose section), and were reimbursed for their participation. The study was approved by the University of Wollongong and Illawarra and Shoalhaven Local Health District Health and Medical Human Research Ethics Committee and registered as a clinical trial (ISRCTN89498802).

### Procedure, CBD administration, and dose

Further detail regarding the methodology of this pragmatic trial is provided by Solowij et al. (companion article^38^). In brief, participants attended two comprehensive testing sessions at the beginning (baseline) and completion (post-treatment) of ∼10 weeks of CBD administration, involving MRI scanning and other assessments (electroencephalography, neuropsychological, and clinical, not reported in this study). Participants were also required to attend brief weekly appointments throughout the trial for monitoring of physiological and psychological well-being, collection of blood and urine samples, and provision of CBD capsules. Participants were not asked to make any changes to their cannabis use patterns during the trial, but were instructed to abstain from cannabis, alcohol, and other substances for a minimum 12 h before baseline and post-treatment testing sessions. Participants were requested to abstain from use of illicit substances other than cannabis throughout the trial and were advised urine drug screens would be conducted to corroborate their self-report.^38^

At each weekly session, participants were provided 28 gelatin-coated capsules for oral administration containing 50 mg of 99.5% pure crystalline CBD (of herbal origin) solved in Miglyol 812 and Softisan 378 (Trigal Pharma Ltd.; BioSynthesis Pharma Group Ltd.). Participants were instructed to consume four capsules per day (100 mg in the morning and 100 mg in the evening, totaling 200 mg daily). This was selected as a “medium” dose in accordance with the range of therapeutic doses reported in human studies (e.g., 800–1000 mg/day well tolerated for up to 6 weeks in psychotic individuals^39,40^), as well as for caution as no previous study had administered prolonged and relatively high doses of CBD to current cannabis users. Participants received an SMS text message each morning and evening reminding them to consume their capsules. At each weekly session, participants returned any unused capsules. Adherence was measured by participants self-reporting the times of any missed doses, using a Timeline Follow-Back Procedure,^41^ and corroborated by the number of capsules returned.

### Measures

Participants' demographic data were obtained and lifetime substance use quantified through structured interview. The Timeline Follow-Back Procedure^41^ was employed at baseline and each subsequent session to obtain a specific and accurate history of substance use throughout the trial (cumulative cannabis, alcohol, and tobacco use across the trial quantified). Cannabis use was converted to standardized units of “cones” (1 joint=3 cones; https://cannabissupport.com.au/media/1593/timeline-followback.pdf) for quantification. The Alcohol Use Disorders Identification Test,^42^ Severity of Dependence Scale,^43^ and Cannabis Withdrawal Scale^44^ were administered at baseline and post-treatment to assess extent of alcohol consumption, severity of cannabis dependence, and cannabis withdrawal symptoms from abstaining before the test session. Blood and urine samples were collected at each session to enable assay of CBD, THC, and THC metabolite concentrations in plasma^45^ (following trial completion), and for corroboration of self-reported substance use by urine toxicology.

Vocabulary and matrix reasoning subscales of the Wechsler Abbreviated Scale of Intelligence^46^ were administered at baseline to estimate full scale intelligence quotient (IQ). Psychological symptoms of depression, anxiety, and psychosis-proneness were assessed at baseline and post-treatment using the following standardized measures: Beck Depression Inventory^47^; State Trait Anxiety Index^48^; Community Assessment of Psychic Experiences^49^; and Cannabis Experiences Questionnaire (CEQ).^50^ Alternate forms of the Rey Auditory Verbal Learning Test (RAVLT),^51^ demonstrated to be sensitive to cannabis-related memory impairment,^52,53^ were also administered at baseline and post-treatment.

### MRI acquisition and processing

**FIG. 1.**
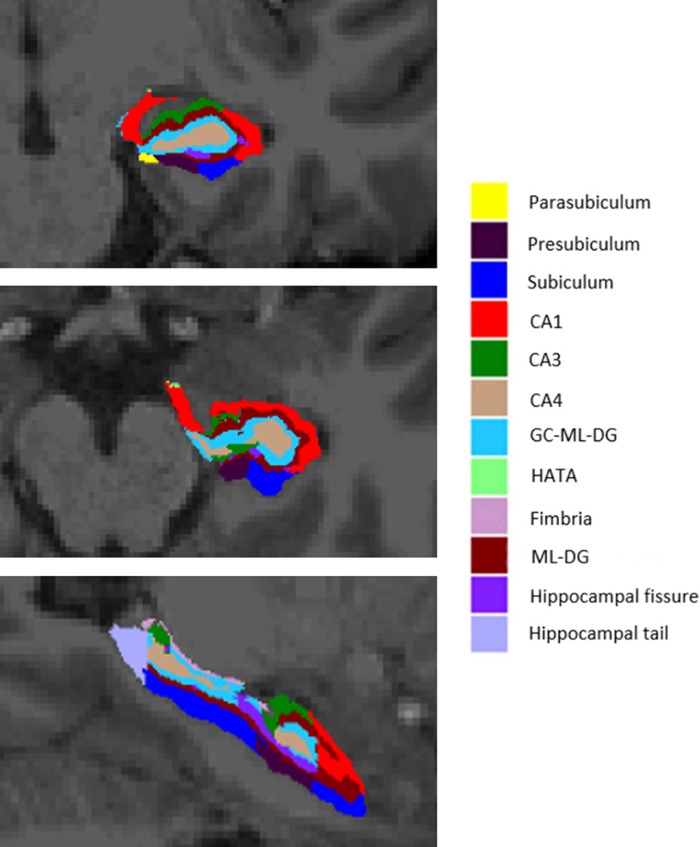
Cross-sectional slices of a T1-weighted image from one participant with automated segmentation of hippocampal subfields overlaid. Coronal (top), axial (middle), and sagittal (bottom) views. CA, cornu ammonis; GC-ML-DG, granule cells in the molecular layer of the dentate gyrus; HATA, hippocampal-amygdala transition area; ML-DG, molecular layer of the dentate gyrus.

Participants underwent structural MRI scans on a 3T Siemens Skyra with a 48-channel head and neck coil using a T1-weighted gradient echo sequence, MPRAGE, with 900 ms inversion time, TR/TE of 2300/2.1 ms, FA=9, 192 slices 1 mm thick, field of view=256×256 mm, and matrix=256×256, resulting in 1.0 mm isotropic resolution and a total acquisition time of 5 min and 26 sec. Participants also underwent other MRI scan sequences not reported in this study. T1-weighted images were processed using an automated longitudinal hippocampal subfield segmentation protocol available through FreeSurfer neuroimaging software version 6.0 (https://surfer.nmr.mgh.harvard.edu).^54^ This technique relies upon the creation of an unbiased subject-specific atlas, which each within-subject time point is then reprocessed against, to reduce within-subject variability.^55^ Furthermore, it has demonstrated greater sensitivity in identifying subtle hippocampal subregional changes than previous cross-sectional processing methods.^54^ Images were first processed through the longitudinal FreeSurfer stream,^55^ involving motion correction, skull stripping, intensity normalization, Talairach transformation, atlas registration, estimation of total intracranial volume, segmentation of white and gray matter volumes, and parcellation of subcortical structures, including the hippocampus. Hippocampal segmentation was then applied, generating volumes for whole left and right hippocampi as well as 12 subfields for each (parasubiculum, presubiculum, subiculum, CA1, CA2/3, CA4, granule cells in the molecular layer of the dentate gyrus [GC-ML-DG], hippocampal-amygdala transition area, fimbria, molecular layer of the dentate gyrus [ML-DG], hippocampal fissure, and hippocampal tail), as depicted in Figure 1.

### Statistical analyses

Statistical analyses were performed using SPSS version 21.0. Paired *t*-tests were conducted to assess volumetric change from baseline to post-treatment for total left and right hippocampal volumes and for the 12 subfields comprising each. As this was a novel exploratory pilot study in a small sample with no placebo control, no adjustment was made for multiple comparisons. Correlations examined associations between change in hippocampal subregion volume (post-treatment minus baseline volume, in mm^3^) and plasma CBD concentration, substance use parameters, psychological symptom, and cognitive measures. Outcomes were further explored by group (heavy versus light users, formulated by median split on lifetime cannabis use) using repeated-measures analysis of covariance (ANCOVA).

## Results

Demographic, substance use, and psychological symptom characteristics of the sample are summarized in Table 1. Participants were predominantly young adult males (median age 25 years; four females), most of whom had completed some tertiary education. Participants had used cannabis regularly for a median of 5.17 years and at baseline were smoking cannabis a median of 25.75 days per month. Cannabis frequency and quantity measures did not significantly differ from baseline to post-treatment, indicating that a consistent level of cannabis use was maintained by participants throughout the trial. Most participants refrained from other illicit drug use during the trial^38^ (the one exception was not an outlier on any hippocampal measures and his data were retained in the analyses reported in this study). Median CBD capsule adherence was 93.16% (range 68.67–99.35%). Participants reported experiencing less euphoria when smoking cannabis (lower CEQ Euphoria scores) at post-treatment compared to baseline (*p*=0.001). Changes in other psychological symptoms and cognition from baseline to post-treatment in this sample are provided in Table 1 and reported in detail in a slightly larger sample by Solowij et al.^38^

**Table 1.** **Participant Demographic Data, Substance Use Measures, and Psychological Symptoms at Baseline and Post-Treatment**

	Baseline	Post-treatment	*t*/Z^a^	*p*	Effect size (*d*/*r*)
Age (years)	25.07 [20.56–46.83]	—			
Gender (male/female)	14/4	—			
Handedness (left/right)	2/16	—			
Education (years)	15.50 [11.00–17.50]	—			
IQ	114.44 (9.50)	—			
BMI	22.68 (2.96)	—			
Alcohol frequency (days/month)	3 [0–14]	6 [0–21]	−1.95	0.051^†^	0.33
Alcohol quantity (standard drinks/month)	15.50 [0–102]	27.50 [0–127.50]	−1.22	0.222	0.20
Cumulative alcohol quantity across the trial (standard drinks)	—	97.54 (75.76)			
AUDIT	7.83 (5.10)	7.56 (4.97)	0.57	0.579	0.13
Regular smoker (yes/no)	8/10	—			
Tobacco frequency (days/month)	0.50 [0–30]	5.50 [0–30]	−1.48	0.138	0.25
Tobacco quantity (cigarettes/month)	2.50 [0–300]	8.75 [0–225]	−0.66	0.508	0.11
Cannabis use					
Age of first use (years)	17.09 (2.18)	—			
Age of onset regular use (years)	19.76 (2.11)	—			
Duration regular use (years)	5.17 [0.56–28.83]	—			
Estimated lifetime occasions of use (days)	1734 [141–8708]	—			
Past month frequency (days/month)	25.75 [2–30]	30.00 [3–30]	−1.67	0.096^†^	0.28
Past month quantity (cones)	177.50 [9–1125.00]	165 [8–1080.00]	−0.60	0.551	0.10
Cumulative quantity across the trial (cones)	—	438 [9.50–2195]			
SDS	3.33 (2.47)	3.11 (2.19)	0.56	0.586	0.13
BDI	2 [0–14]	0.50 [0–12]	−2.18	0.029^*^	0.36
STAI state	25.72 (5.15)	29.39 (8.24)	−2.53	0.022^*^	0.67
STAI trait	32 [20–63]	32 [20–49]	−1.52	0.129	0.25
CAPE total frequency	59.83 (10.18)	56.67 (9.13)	1.99	0.063^†^	0.47
CAPE total distress	23.78 (16.40)	19.39 (12.68)	2.09	0.052^†^	0.54
CEQ Euphoria	44.28 (7.78)	38.94 (6.81)	3.90	0.001^*^	0.93
RAVLT words recalled trials 1–5	52.59 (11.52)	55.41 (9.96)	−1.88	0.079^†^	0.47

Mean (SD) or median [range].

^*^ *p*<0.05.

^†^ Trend-level significance.

^a^ Paired samples *t*-test for normally distributed data; Wilcoxon signed-rank test for nonparametric data.

AUDIT, Alcohol Use Disorders Identification Test^42^; BDI, Beck Depression Inventory^47^; BMI, body mass index; CAPE, Community Assessment of Psychic Experiences^49^; CEQ, Cannabis Experiences Questionnaire^50^; IQ, intelligence quotient; SD, standard deviation; SDS, Severity of Dependence Scale^43^; STAI, State Trait Anxiety Index^48^; RAVLT, Rey Auditory Verbal Learning Task.^51^

Mean and significance values of all hippocampal volumetric comparisons are presented in Table 2. Paired samples *t*-tests revealed no significant change in total left or right hippocampal volume, nor in nine hippocampal subfields. However, a significant increase in left parasubiculum volume was observed (*p*=0.035), with trend-level increases in the left presubiculum (*p*=0.058) and left subiculum (*p*=0.066). In addition, a trend toward decreased volume in the right presubiculum was found (*p*=0.056). Subsequently, to increase reliability, total volumes of all subicular subfields (parasubiculum, presubiculum, and subiculum) were pooled to create a “subicular complex”^56,57^ for each hemisphere. Paired samples *t*-tests showed a significant increase in left subicular complex volume (*p*=0.017; Cohen's *d*=0.63), but no change in the right subicular complex (*p*=0.471; Cohen's *d*=0.17). Changes in left and right subicular complex and substructures are detailed in Table 3.

**Table 2.** **Hippocampal Whole and Subfield Volumes (mm^3^) at Baseline and Post-Treatment**

	Baseline	Post-treatment	*p*
Whole hippocampus			
Left	3940.65 (333.06)	3962.13 (344.81)	0.180
Right	4127.15 (356.02)	4119.00 (375.98)	0.682
Subicular complex			
Left	885.18 (81.89)	899.17 (85.15)	0.017^*^
Right	865.83 (99.15)	862.06 (102.53)	0.471
Parasubiculum			
Left	64.36 (9.79)	66.18 (10.57)	0.035^*^
Right	64.97 (12.77)	64.29 (12.11)	0.418
Presubiculum			
Left	342.48 (24.29)	347.77 (24.62)	0.058^†^
Right	334.71 (44.03)	330.36 (46.80)	0.056^†^
Subiculum			
Left	478.33 (60.32)	485.22 (62.14)	0.066^†^
Right	466.15 (49.03)	467.40 (51.55)	0.697
CA1			
Left	706.73 (73.16)	710.41 (77.20)	0.322
Right	750.62 (73.80)	754.35 (78.14)	0.380
CA2/3			
Left	252.73 (42.57)	254.05 (42.93)	0.371
Right	284.33 (33.23)	283.08 (34.48)	0.533
CA4			
Left	326.63 (41.35)	326.89 (40.76)	0.911
Right	353.30 (46.98)	352.50 (49.61)	0.760
GC-ML-DG			
Left	360.98 (40.63)	360.80 (40.24)	0.935
Right	383.47 (45.14)	383.72 (48.72)	0.931
HATA			
Left	62.54 (8.67)	62.76 (8.97)	0.812
Right	65.30 (6.82)	64.78 (7.02)	0.359
Fimbria			
Left	96.37 (15.78)	97.26 (15.77)	0.482
Right	92.46 (23.75)	92.11 (23.58)	0.848
ML-DG			
Left	667.82 (61.27)	670.34 (61.26)	0.450
Right	695.79 (60.06)	698.67 (64.04)	0.413
Hippocampal fissure			
Left	155.92 (27.44)	157.35 (21.56)	0.725
Right	156.50 (18.92)	152.37 (20.76)	0.379
Hippocampal tail			
Left	581.66 (62.20)	580.44 (63.11)	0.777
Right	636.05 (71.03)	627.74 (66.95)	0.126

Mean (SD) and paired *t*-test significance values. Subicular complex is the sum of parasubiculum, presubiculum, and subiculum volumes.

^*^ *p*<0.05.

^†^ Trend-level significance.

CA, cornu ammonis; GC-ML-DG, granule cells in the molecular layer of the dentate gyrus; HATA, hippocampal-amygdaloid transition area; ML-DG, molecular layer of the dentate gyrus.

**Table 3.** **Percentage Change in Volume from Baseline to Post-Treatment and Effect Sizes for Left and Right Subicular Complex and Substructures**

	% change	Effect size (*d)*
Left subicular complex	1.58	0.63
Left parasubiculum	2.83	0.55
Left presubiculum	1.54	0.48
Left subiculum	1.44	0.47
Right subicular complex	−0.44	0.17
Right parasubiculum	−1.05	0.20
Right presubiculum	−1.30	0.51
Right subiculum	0.27	0.10

% change calculated as ([post-treatment volume–baseline volume]/baseline volume)×100.

Correlations did not reveal any significant association between left subicular complex or subregion volume change and plasma CBD concentration, cumulative CBD dose (calculated by number of capsules returned by participants), or weeks of CBD treatment. Right subicular complex volumetric change was significantly correlated with plasma CBD concentration in the final trial week (*ρ*=0.62, *p*=0.006), as well as a trend-level association with mean CBD plasma concentration over the trial (*ρ*=0.45, *p*=0.064), suggesting that higher plasma CBD was associated with increased growth in this area, despite the overall tendency toward a decrease in this region from baseline to post-treatment (Table 3). Age was not significantly correlated with any subicular change, other than at trend level in the left parasubiculum (*ρ*=−0.43, *p*=0.073), indicating growth in this subregion was more pronounced in younger participants, potentially implicating a role of brain maturation. Subicular complex or subregion volume change did not significantly correlate with any substance use measures; importantly, change in left subicular complex volume was not associated with changes in alcohol use frequency or quantity from baseline to post-treatment (both *p*>0.601), nor with cumulative quantity of cannabis, alcohol, or cigarettes used over the trial (all *p*>0.114). Left subiculum volume was correlated with lifetime duration of cannabis use at baseline (*ρ*=−0.54, *p*=0.021; trend for left subicular complex volume *ρ*=−0.44, *p*=0.066), suggesting that smaller volumes at baseline were associated with greater cannabis exposure, and these normalized with CBD treatment. A significant inverse correlation was observed between change in right subiculum volume and change in desired level of intoxication when smoking cannabis (scale 1–10 [what level of intoxication do you usually like to reach?], which tended to decrease from baseline to post-treatment: 7.14 vs. 6.97, respectively; *r*=−0.51, *p*=0.031; trend level also for left parasubiculum, *r*=−0.43, *p*=0.074; and left subicular complex, *r*=−0.42, *p*=0.080). This suggests that increased volume in these subicular regions was associated with lesser desired level of “high” following CBD treatment. No significant correlations between subicular complex or subregion volumes and psychological symptom or cognitive (RAVLT) measures were found.

**FIG. 2.**
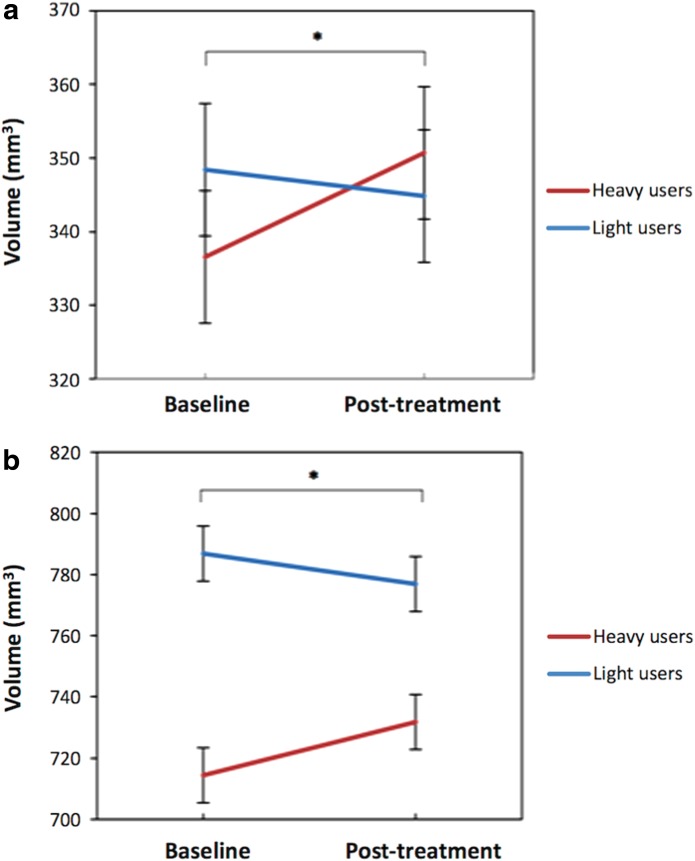
Time by group interaction for **(a)** left presubiculum (*p=0.015) and **(b)** right CA1 (*p=0.012) volume, controlling for intracranial volume and cumulative cannabis use (cones smoked) across the trial. Error bars represent standard error.

Hippocampal volume change was further explored between and within groups of heavy and light users (median split on lifetime occasions of use, *n*=9 [seven male, eight right handed] each). Group differences in cannabis use measures are presented in Table 4. Heavy and light users significantly differed on lifetime cannabis use, frequency and quantity of cannabis use at baseline and post-treatment, cumulative cones smoked across the trial, as well as years of education (median years: heavy=12.50 and light=16.00; *p*=0.003), but did not differ in age (*p*=0.070) or IQ (*p*=0.056), any plasma CBD concentration measure (all *p*>0.063), nor in alcohol or tobacco use or change over the trial (all *p*>0.136). Years of education was not correlated with any hippocampal volume measure, so was not included as a covariate in analyses. Heavy and light users did not significantly differ on any hippocampal total or subregion volume measure at baseline, other than at trend level for the left fimbria (*p*=0.056). Repeated-measures ANCOVAs were conducted with group as a between-subjects factor and intracranial volume and cumulative cones smoked across trial as covariates. As reported in Table 5, no significant main effects of time were found for any total hippocampal or subregion volume. However, significant time by group interactions were observed in the left presubiculum (*p*=0.015) and right CA1 (*p*=0.012). Figure 2a and 2b depict a marked increase in volume in these subregions from baseline to post-treatment in heavy users, yet a slight decrease in lighter users, with both groups showing more similar volume post-treatment. *Post hoc* repeated measures ANCOVAs (controlling for cumulative cannabis use across the trial) were performed within heavy and light user groups separately. Significant main effects of time were found only in heavy users for the left presubiculum (*p*<0.001; light users *p*=0.80), left subicular complex (*p*=0.003; light users *p*=0.393), and trend level for left subiculum (*p*=0.064; light users *p*=0.344), indicating that the overall finding of increased left subicular complex volume across the trial was driven by the heavy user group. In addition, a significant main effect of time was observed in heavy users for the right CA1 (*p*=0.036; light users *p*=0.577).

**Table 4.** **Heavy and Light User Group Differences in Cannabis Use Measures**

	Heavy (*n*=9)	Light (*n*=9)	*Z*	*p*
Duration regular use (years)				
Baseline	6.53 [4.13–28.83]	3.39 [0.56–5.53]	−3.18	<0.001^*^
Estimated lifetime occasions of use (days)				
Baseline	2608.50 [1734.00–8707.91]	452.00 [141.00–1448.00]	−3.58	<0.001^*^
Past month frequency (days/month)				
Baseline	30.00 [9.00–30.00]	12.00 [2.00–30.00]	−2.68	0.006^*^
Post-treatment	30.00 [13.00–30.00]	16.00 [3.00–30.00]	−2.62	0.010^*^
Past month quantity (cones)				
Baseline	375.00 [75.00–1125.00]	42.00 [9.00–463.75]	−2.48	0.011^*^
Post-treatment	472.50 [58.50–1080.00]	55.00 [8.00–600.00]	−2.56	0.008^*^
Cumulative quantity across the trial (cones)				
Post-treatment	1282.00 [317.00–2195.00]	113.00 [9.50–815.00]	−2.96	0.002^*^

Median [range] and Wilcoxon signed-rank test significance values.

^*^ *p*<0.05.

**Table 5.** **Adjusted Mean (Standard Error) of Hippocampal Whole and Subfield Volumes for Heavy and Light User Groups**

	Heavy (*n*=9)		Light (*n*=9)			
	Baseline	Post-treatment	Baseline	Post-treatment	Time *p*	Time by group *p*
Whole hippocampus						
Left	3822.57 (60.57)	3872.15 (62.23)	4058.73 (60.57)	4052.10 (62.23)	0.197	0.198
Right	4025.95 (71.47)	4036.63 (68.91)	4228.35 (71.47)	4201.36 (68.91)	0.157	0.507
Subicular complex						
Left	857.42 (20.96)	884.06 (22.77)	912.94 (20.96)	914.28 (22.77)	0.683	0.104
Right	871.28 (22.48)	865.40 (20.59)	860.38 (22.48)	858.71 (20.59)	0.254	0.780
Parasubiculum						
Left	61.74 (3.30)	62.71 (3.74)	66.98 (3.30)	69.64 (3.74)	0.591	0.471
Right	66.50 (2.69)	65.56 (2.16)	63.44 (2.69)	63.03 (2.16)	0.195	0.322
Presubiculum						
Left	336.55 (9.00)	350.70 (9.00)	348.41 (9.00)	344.83 (9.00)	0.752	0.015^*^
Right	336.50 (14.10)	334.65 (14.92)	332.93 (14.10)	326.07 (14.92)	0.341	0.436
Subiculum						
Left	459.13 (15.99)	470.64 (16.76)	497.54 (16.76)	499.80 (16.76)	0.612	0.375
Right	468.29 (9.99)	465.20 (8.04)	464.01 (9.99)	469.61 (8.04)	0.977	0.829
CA1						
Left	678.61 (15.72)	686.20 (16.18)	734.85 (15.72)	734.63 (16.18)	0.064^†^	0.424
Right	714.39 (15.91)	731.81 (18.79)	786.84 (15.91)	776.89 (18.79)	0.119	0.012^*^
CA2/3						
Left	238.72 (12.66)	240.03 (12.30)	266.74 (12.66)	268.08 (12.30)	0.390	0.995
Right	273.97 (9.03)	271.91 (8.77)	294.69 (9.03)	294.25 (8.77)	0.347	0.788
CA4						
Left	313.37 (10.03)	313.59 (9.16)	339.90 (10.03)	340.20 (9.16)	0.409	0.990
Right	333.44 (12.77)	334.74 (13.22)	373.17 (12.77)	370.26 (13.22)	0.140	0.573
GC-ML-DG						
Left	346.29 (9.99)	347.79 (9.24)	375.68 (9.99)	373.81 (9.24)	0.400	0.614
Right	364.66 (11.43)	368.37 (11.87)	402.29 (11.43)	399.07 (11.87)	0.085^†^	0.378
HATA						
Left	59.46 (2.85)	60.61 (3.46)	65.63 (2.85)	64.92 (3.46)	0.486	0.453
Right	63.67 (2.19)	62.73 (2.46)	66.94 (2.19)	66.83 (2.46)	0.523	0.631
Fimbria						
Left	88.24 (4.83)	88.38 (4.77)	104.50 (4.83)	106.14 (4.77)	0.836	0.697
Right	86.02 (8.33)	81.41 (7.80)	98.90 (8.33)	102.81 (7.80)	0.201	0.055^†^
ML-DG						
Left	647.81 (13.35)	655.02 (12.26)	687.84 (13.35)	685.65 (12.26)	0.164	0.304
Right	679.17 (11.95)	689.94 (13.56)	712.41 (11.95)	707.41 (13.56)	0.224	0.106
Hippocampal fissure						
Left	160.18 (10.97)	157.78 (9.29)	151.66 (10.97)	156.91 (9.29)	0.365	0.501
Right	159.09 (8.39)	149.02 (8.84)	153.92 (8.39)	155.72 (8.84)	0.159	0.370
Hippocampal tail						
Left	592.66 (25.66)	596.47 (25.11)	570.66 (25.66)	564.41 (25.11)	0.661	0.426
Right	639.38 (29.88)	630.33 (27.22)	632.72 (29.88)	625.14 (27.22)	0.416	0.926

Repeated measures ANCOVA main effect of time and time by group interactions, controlling for intracranial volume and cumulative cannabis use (cones smoked) across the trial. Subicular complex is the sum of parasubiculum, presubiculum, and subiculum volumes.

^*^ *p*<0.05.

^†^ Trend-level significance.

**FIG. 3.**
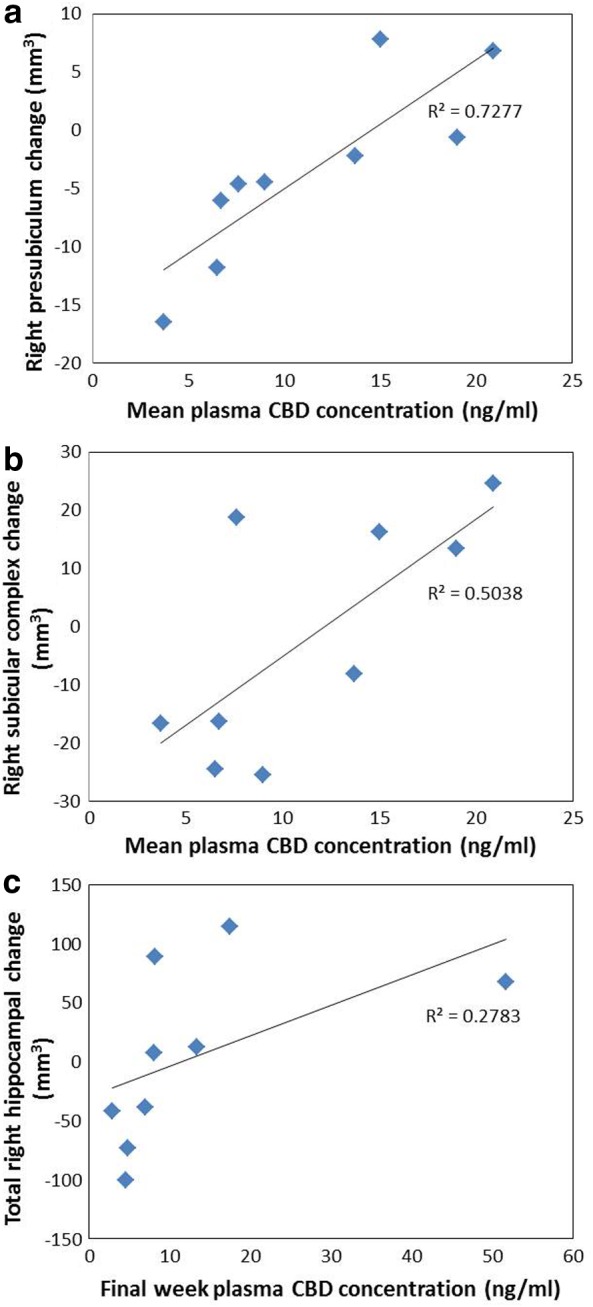
Significant associations in heavy users between **(a)** mean plasma CBD concentration and right presubiculum volume change; **(b)** mean plasma CBD concentration and right subicular complex volume change; and **(c)** final week CBD plasma concentration and right total hippocampal volume change. CBD, cannabidiol.

Exploratory correlations within heavy and light user groups separately indicated associations with mean, maximum, and final trial week plasma CBD concentrations in heavy users only, for the right subicular complex (CBD plasma: mean, *r*=0.71, *p*=0.032; maximum, *r*=0.68, *p*=0.045; and final week, *ρ*=0.82, *p*=0.007), right presubiculum (mean, *r*=0.85, *p*=0.003; maximum, *r*=0.69, *p*=0.040; and final week, *ρ*=0.70, *p*=0.036), and right subiculum (final week, *ρ*=0.68, *p*=0.042), indicating greater positive change (increased volumes) with higher CBD concentrations (Fig. 3a, b). In heavy users, final week CBD plasma was also correlated with change in the whole right hippocampus (*ρ*=0.87, *p*=0.002, as shown in Fig. 3c; *p*=0.002 also with removal of outlier) and in the right GC-ML-DG (*ρ*=0.70, *p*=0.036), and trend level with right CA1 change (*ρ*=0.63, *p*=0.067), suggesting that growth in these areas may be CBD treatment related.

## Discussion

This study reports outcomes from the first trial of prolonged CBD treatment to cannabis users within the community. We performed an automated longitudinal hippocampal subfield segmentation to examine the potential for chronic CBD administration to restore characteristic hippocampal volumetric reduction in regular cannabis users. We found an overall significant increase of left subicular complex volume from baseline to post-treatment, subserved by growth in the left parasubiculum, presubiculum, and subiculum, with medium-large effect sizes. We compared heavy and light users to explore the influence of prior cannabis exposure on CBD treatment outcomes, finding that the increased left subicular complex volume was driven by heavy users, particularly the marked growth that occurred in the left presubiculum, which normalized post-treatment volumes toward those observed in light users. Similarly, only heavy users showed a significant increase in right CA1 volume over the trial. Despite an overall trend toward decreased right presubiculum volume from baseline to post-treatment, significant associations between higher plasma CBD concentration and increased growth in this region were apparent, particularly in heavy users. Plasma CBD concentration was also significantly correlated with right subicular complex and total right hippocampal growth in heavy users.

These findings suggest a regionally specific restorative effect of CBD upon the subicular and CA1 subfields for heavy cannabis users, in the context of ongoing and relatively consistent cannabis use during the trial. The subicular subregions receive projections from CA1 and function as the primary hippocampal output structures, interfacing with the entorhinal cortex and a range of cortical and subcortical sites.^58^ Functionally, CA1 neurons are critically involved in the representation of temporal and spatial contextual information and retrieval of episodic memory,^59,60^ while a dorsal-ventral functional segmentation has been suggested for subicular subregions, whereby dorsal subiculum is involved in spatial representation and memory and ventral subiculum is involved in hypothalamic–pituitary–adrenal regulation of stress, anxiety, and reward.^61^

Subicular and CA1 subregions are implicated in pathophysiological models of various conditions.^62^ For example, atrophy of the presubiculum, subiculum, and CA1 are the earliest sites of hippocampal degeneration in Alzheimer's disease^62–64^; subiculum, presubiculum, and right CA1 volume reduction have been demonstrated in patients with schizophrenia^65^ and bipolar disorder;^65–67^ and subiculum and CA1 shape alterations have been found in patients with major depressive disorder^68,69^ and schizophrenia^70^ relative to healthy controls. Substantial evidence has revealed a pattern of progressive hippocampal pathophysiology in patients with schizophrenia, beginning with extracellular glutamate dysregulation driving hypermetabolism in CA1, which precipitates attenuated psychotic symptoms, and extends to the subiculum during the transition to frank psychosis.^71–73^ Longitudinal studies in ultra-high-risk patients have demonstrated focal atrophy of CA1, at a rate of 6% per year, differentiates those who progress onto syndromal psychosis and those whose attenuated psychotic symptom remit, with an extension of atrophy to the subiculum then other subfields as the illness progresses.^73,74^ The rate of CA1 volume reduction specifically was found to predict worsening of symptom severity over time,^74^ highlighting the importance of preventing degeneration in this subregion. Furthermore, in schizophrenia patients, subiculum volume reduction has been associated with increased negative symptoms,^65^ and left presubiculum atrophy has been correlated with impairments in episodic memory.^75^

Interestingly, 12 weeks of antipsychotic treatment was found to increase subicular (but not CA1) volume (encompassing all subicular subregions) by an average 32 mm^3^ (2.55%) in patients experiencing a first-episode psychosis, while a healthy young adult control group (of a similar age to the sample reported in this study; mean [standard deviation]: 22.2 [4.9]) showed negligible change in this region (−0.53%) over the same period.^76^ This suggests natural fluctuation in subicular volume or the influence of ongoing brain maturation across our similar trial length and participant sample is unlikely, supporting a CBD treatment effect. Given the parallels observed in schizophrenia pathophysiology and neurobiological changes conferred by chronic cannabis use,^77,78^ and interest in the antipsychotic properties of CBD,^25^ our finding of volumetric restoration in the subicular and CA1 regions is promising, and this therapeutic indication for CBD may extend to benefitting other clinical groups (e.g., schizophrenia).

The greater volumetric increase in heavy cannabis users is consistent with psychological symptom and cognitive outcomes from this trial, showing that dependent users experienced a significantly greater reduction in depressive and psychotic-like symptoms and improved cognition compared to nondependent users (reporting on *n*=20 participants^38^). In addition, we recently demonstrated protective effects of chronic CBD treatment against the development of negative psychotic-like symptoms in a preclinical model of schizophrenia, with no effect in control animals.^79^ While a ceiling effect may have occurred in lighter users, our findings nevertheless provide further support for the contention that CBD may confer greater therapeutic effects in a more compromised brain.

Our findings suggest a restorative effect of CBD on hippocampal substructures in cannabis users, even within the context of continued cannabis use. In the absence of ongoing use, as might occur in a motivated treatment-seeking sample, greater neurotherapeutic benefit may be expected. During the trial, participants reported feeling less high after using cannabis and this was corroborated by significant reduction at post-treatment on the CEQ Euphoria subscale. As such, CBD may be a valuable adjunct to psychological treatments for cannabis dependence. Furthermore, subjective ratings of preferred level of cannabis intoxication were negatively associated with increased subicular region growth.

A critical limitation of this study was that it was a pragmatic, open-label trial without a placebo control. While it is unlikely that brain structural measures are amenable to expectancy effects, our findings should nevertheless be interpreted with caution. In addition, the sample was relatively high-functioning, and some participants' cannabis use was infrequent. If therapeutic effects of CBD are more likely to manifest in a disease state, this gives even greater credence to our findings and stronger effects may be expected in a more entrenched sample. As the majority of participants were young adult males, the influence of ongoing brain maturation must be considered and future research should examine age effects as well as potential sex differences in CBD treatment effects. That some hippocampal subfield volume changes were associated with plasma CBD concentrations supports the changes being CBD treatment related. Nevertheless, replication in a larger sample placebo-controlled trial is warranted.

The mechanisms underlying a restorative effect of CBD on hippocampal subfields in cannabis users remain to be elucidated. While neurogenesis is plausible, no change was observed in the DG, the primary hippocampal subregion implicated in neurogenesis. The potential involvement of both CB1Rs and glutamate is worthy of further investigation, given the high density of cannabinoid receptors in the subicular complex and CA1 regions,^15^ and evidence suggesting glutamatergic dysregulation to be a driver of subicular and CA1 volume changes associated with psychosis.^72,73^ The differential pattern of volume change observed across left and right hippocampi is also of interest. While the majority of studies examining cannabis-related hippocampal pathophysiology which have reported hemispheric lateralization effects found volume reduction to be more pronounced in the right hippocampus,^10,80,81^ inverse associations between left hippocampal volume and cannabis exposure (e.g., cumulative use^9^ and quantity per week^82^) have also been reported. Mechanisms for hemispheric differentiation remain unclear, and future research should seek to examine potential lateralization effects in CBD treatment response.

In conclusion, our findings are the first to demonstrate an ameliorating effect of CBD treatment upon brain structural harms characteristic of regular cannabis use. Furthermore, these results speak to the potential for CBD treatment to restore hippocampal pathology in a range of clinical populations (e.g., schizophrenia, Alzheimer's disease, and major depressive disorder).

## Abbreviations Used

ANCOVA: analysis of covariance
AUDIT: Alcohol Use Disorders Identification Test
BDI: Beck Depression Inventory
BMI: body mass index
CA: cornu ammonis
CAPE: Community Assessment of Psychic Experiences
CB1Rs: Cannabinoid type 1 receptors
CBD: cannabidiol
CEQ: Cannabis Experiences Questionnaire
DG: dentate gyrus
GC-ML-DG: granule cells in the molecular layer of the dentate gyrus
HATA: hippocampal-amygdaloid transition area
IQ: intelligence quotient
ML-DG: molecular layer of the dentate gyrus
MRI: magnetic resonance imaging
NAA: n-acetylaspartate
RAVLT: Rey Auditory Verbal Learning Test
SD: standard deviation
SDS: Severity of Dependence Scale
STAI: State Trait Anxiety Index
THC: Δ^9^-tetrahydrocannabinol
